# Crosstalk between NRF2 and HIPK2 shapes cytoprotective responses

**DOI:** 10.1038/onc.2017.221

**Published:** 2017-07-10

**Authors:** L Torrente, C Sanchez, R Moreno, S Chowdhry, P Cabello, K Isono, H Koseki, T Honda, J D Hayes, A T Dinkova-Kostova, L de la Vega

**Affiliations:** 1 Division of Cancer Research, School of Medicine, Jacqui Wood Cancer Centre, James Arrott Drive, Ninewells Hospital and Medical School, University of Dundee, Dundee, Scotland; 2 Developmental Genetics, RIKEN Center for Integrative Medical Sciences (IMS), Yokohama, Japan; 3 Department of Chemistry and Institute of Chemical Biology & Drug Discovery, Stony Brook University, Stony Brook, NY, USA

## Abstract

Homeodomain interacting protein kinase-2 (HIPK2) is a member of the HIPK family of stress-responsive kinases that modulates cell growth, apoptosis, proliferation and development. HIPK2 has several well-characterised tumour suppressor roles, but recent studies suggest it can also contribute to tumour progression, although the underlying mechanisms are unknown. Herein, we have identified novel crosstalk between HIPK2 and the cytoprotective transcription factor NRF2. We show that HIPK2 is a direct transcriptional target of NRF2, identifying a functional NRF2 binding site in the *HIPK2* gene locus and demonstrating for the first time a transcriptional mode of regulation for this kinase. In addition, HIPK2 is required for robust NRF2 responsiveness in cells and *in vivo*. By using both gain-of-function and loss-of-function approaches, we demonstrate that HIPK2 can elicit a cytoprotective response in cancer cells via NRF2. Our results have uncovered a new downstream effector of HIPK2, NRF2, which is frequently activated in human tumours correlating with chemoresistance and poor prognosis. Furthermore, our results suggest that modulation of either HIPK2 levels or activity could be exploited to impair NRF2-mediated signalling in cancer cells, and thus sensitise them to chemotherapeutic drugs.

## Introduction

Homeodomain interacting protein kinase-2 (HIPK2) is a member of the HIPK family of stress-responsive kinases, and it modulates cell proliferation, differentiation, apoptosis and development.^1, 2, 3, 4, 5, 6, 7^ HIPK2 responds to a variety of physiological stresses,^3, 8, 9, 10, 11, 12^ transforming these cues into changes in transcriptional programs, which in turn enables cells to adapt to and survive the original insult. Although HIPK2 is highly regulated at the posttranslational level,^9, 10, 13, 14, 15, 16, 17, 18, 19, 20^ essentially no information exists about its transcriptional regulation.

HIPK2 is considered to be a potential haplo-insufficient tumour suppressor as it can promote apoptosis in response to chemotherapeutic drugs and radiation, mainly by phosphorylating p53 at S46,^1, 21^ which then induces expression of pro-apoptotic genes. Additionally, HIPK2 can also protect cells against genome instability induced by genotoxic agents by promoting DNA damage repair pathways.^17, 22^ Interestingly, accumulating evidence suggests that HIPK2 may also support tumour progression: the expression of HIPK2 is significantly higher in cervical cancer than in healthy tissue^23^ and in aggressive meningiomas (when compared with benign meningiomas), where it positively associates with tumour progression.^24^ HIPK2 is also amplified in pilocytic astrocytoma^25^ and in ovarian and prostate tumours (The Cancer Genome Atlas), and positively associates with cell growth in various cancer cell lines.^25, 26, 27^ These results imply that HIPK2 might play a dual role in cancer depending on context, either acting as a tumour suppressor or facilitating tumour progression. While the pathways involved in the tumour suppressor role of HIPK2 are relatively well understood, the underlying mechanisms mediating its cytoprotective function(s) remain unclear.

NRF2 (nuclear factor erythroid 2 (NF-E2) p45-related factor 2, encoded by *NFE2L2*) is the master regulator of oxidative stress responses, which allows adaptation and survival during stress conditions. NRF2 controls the expression of a battery of genes, which encode antioxidant and drug-metabolising enzymes, as well as drug transporters (for example, HO1, GSTs, NQO1 and MRPs), all of which contain antioxidant response elements (AREs) within their promoter/enhancer regions.^28, 29^ In normal cells, NRF2 activity is kept low under non-stress conditions by its rapid proteasomal degradation, which is principally mediated by KEAP1 (a substrate adaptor for a Cul3-based E3 ubiquitin ligase).^30, 31^ Upon exposure to electrophiles or reactive oxygen species, KEAP1 undergoes conformational changes that impair its substrate adaptor function, leading to the accumulation of newly synthesised NRF2, which can then translocates to the nucleus and activates its target genes.^32^ Furthermore, NRF2 controls the expression of a number of its own regulators (for example, KEAP1, p66, p62), thus creating autoregulatory loops that control the amplitude/duration of its own response.^33, 34, 35^

Although NRF2 is cytoprotective and its transient activation is linked with chemoprevention,^36^ it has become apparent that its sustained activation protects tumour cells against chemo- and radiotherapy and can promote metabolic activities that support cell proliferation and tumour growth.^37, 38, 39, 40^ Not surprisingly, therefore, NRF2 is often constitutively activated in human tumours,^39, 41^ where it is associated with poor prognosis.^42, 43, 44^ This sustained activation of NRF2 is especially relevant in lung tumours, where due to mutations in *KEAP1* or *NFE2L2*, NRF2 is constitutively activated in 30–60% of cases.^41, 45^ Additionally, NRF2 can be upregulated by the oncogenic mutant KRAS, BRAF and Myc and by loss of PTEN,^46, 47^ suggesting that aberrant activation of NRF2 is a common event in many cancer types.

In this study, we describe for the first time the existence of crosstalk between HIPK2 and NRF2 in which NRF2 regulates HIPK2 expression, and in turn, HIPK2 positively shapes the NRF2 response.

## Results and discussion

### NRF2 transcriptionally regulates basal and inducible levels of HIPK2

**Figure 1:**
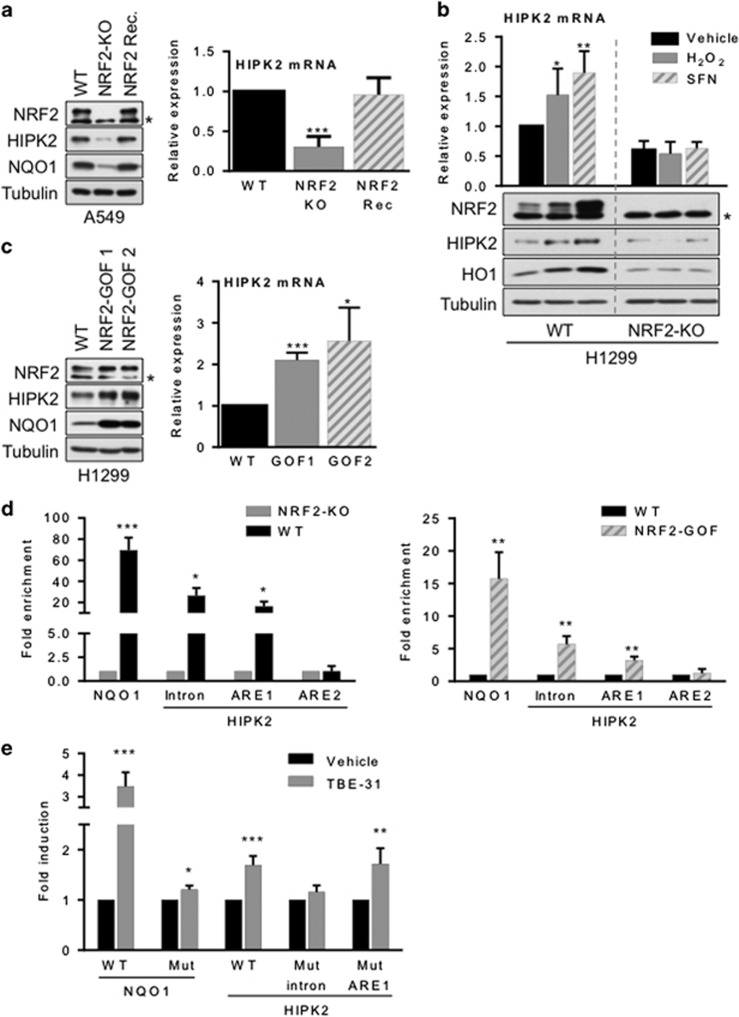
HIPK2 is a novel NRF2 target gene. (**a**) All cell lines used in the study have been validated by STR profiling and were routinely tested for mycoplasma. When applicable, the differences between groups were determined by unpaired Student’s *t*-test. Analyses were performed using GraphPad Prism (GraphPad Software Inc., La Jolla, CA, USA); a *P*-value of <0.05 was considered significant. *P⩽0.05, **P⩽0.01, ***P⩽0.001. The endogenous *NFE2L2* gene, which encodes NRF2, was edited by transfecting cells with pLentiCRISPR-v2 (a gift from Dr Feng Zhang, Addgene plasmid #52961) containing a guide RNA (gRNA) directed against the KEAP1-binding domain within the *NFE2L2* locus (5′-TGGAGGCAAGATATAGATCT-3′). CRISPR-mediated gene editing with this gRNA produced NRF2 knockout clones (NRF2-KO), and NRF2 gain-of-function clones (NRF2-GOF). NRF2-GOF clones were those that the Cas9-mediated cleavage was repaired in frame but introducing mutations (deletions or insertions) within the KEAP1-binding domain. After 2 days of puromycin selection, cells were clonally selected by serial dilution, and positive clones were identified as previously described.^66^ Control cells (referred as wild-type) comprises the pooled population of surviving cells transfected with pLentiCRISPRv2 vector (empty backbone) treated with puromycin. Mutational gene changes in NFE2L2 were validated by sequencing of their genomic DNA. All our results were validated using at least two different clones from each gRNA. Lentiviral infections were performed as previously described.^10^ Left panel: A549 cells were grown in DMEM (Thermo Fisher Scientific, Waltham, MA, USA) containing 10% fetal bovine serum (FBS). A549 control cells (WT) infected with empty vector were compared with CRISPR-mediated NRF2 knockout cells (NRF2-KO) infected with empty vector or with lentivirus encoding NRF2 (NRF2 Rec). Protein levels of human NRF2 (ab62352, Abcam, Cambridge, UK), HIPK2^6^ and NQO1^67^ were analysed by western blotting. Tubulin was used as a loading control (TU-02, Santa Cruz Biotechnology, Dallas, TX, USA). Note that antibodies against human NRF2 recognise a non-specific protein as a faster migrating band; such band has been previously described.^68, 69, 70^ The asterisk marks the position of the non-specific band. Right panel: TaqMan analyses of HIPK2 mRNA levels in A549 control cell lines (WT) compared with NRF2-KO cells or NRF2-KO cells reconstituted with NRF2 (NRF2 Rec). The data were normalised using β-actin as an internal control. The mRNA levels in WT cells were set as 1. Values are means plus s.d. from three independent experiments. RNA was extracted using RNeasy kit (Qiagen, Hilden, Germany), and reverse-transcribed to complimentary DNA (cDNA) using Omniscript RT kit (Qiagen) according to the manufacturer’s instructions. Resulting cDNA was analysed using TaqMan Universal Master Mix II (Life Technologies, Carlsbad, CA, USA). Gene expression was determined using an Applied Biosystems (Foster City, CA, USA) 7300 Real-Time PCR system by the comparative ΔΔCT method. The following primers and probes were used in the study: hHIPK2-F 5′-CATGAAGCAGAGACAGGGAT-3′, hHIPK2-R 5′-CATCAATGGTCAGCATCTTC-3′, hHIPK2-Probe 5′-GATGATATGGCCCAGGTGA-3′, hActin-F 5′-GCGCGGCTACAGCTTCA-3′, hActin-R 5′-TCTCCTTAATGTCACGCACGAT-3′, hActin-Probe 5′-CACCACGGCCGAGCGGGA-3′. (**b**) WT or NRF2-KO H1299 were treated with vehicle, 100 μm hydrogen peroxide (H_2_O_2_) or with 3 μm Sulforaphane (SFN). After 3 h, cells were lysed and subjected to western blotting or mRNA analyses (RT-qPCR). Upper panel: levels of HIPK2 mRNA were analysed. The mRNA levels in vehicle-treated WT cells were set as 1. Values are means plus s.d. from five independent experiments. Sulforaphane [1-isothiocyanato-4(R,S)-(methylsulfinyl)butane] was obtained from LKT Labs (St. Paul, MN, USA). Lower panel: the levels of the indicated proteins were analysed. HO1 antibody was purchased from Biovision (Biovision Inc., Milpitas CA, USA). (**c**) H1299 control cells (WT) were compared with CRISPR-mediated NRF2 gain-of-function (NRF2-GOF) H1299 cells. Results obtained using two independent NRF2-GOF clones are shown. Left panel: the levels of the indicated proteins were analysed. Right panel: Taqman analysis of HIPK2 mRNA levels. (**d**) ChIP analysis of NRF2 occupancy within the HIPK2 proximal promoter (ARE1 and ARE2), HIPK2 intronic region (Intron) and the previously characterised ARE within the NQO1 promoter.^71^ ChIp analyses were performed as previously described^71^ using the following primers: NQO1-ARE-F 5′-CCCTTTTAGCCTTGGCACGAAA-3′, NQO1-ARE-R 5′-TGCACCCAGGGAAGTGTGTTGTAT-3′, HIPK2-ARE-Intron-F 5′-GTCCCATTATACCTTCGCAG-3′, HIPK2-ARE-Intron-R 5′-AGCATGTCCACAGAGCCTC-3′, HIPK2-ARE1-F 5′-GCGTGCACACACACACACAAAG-3′, HIPK2-ARE1-R 5′-GGAAGGCCGAACCGAAGGG-3′, HIPK2-ARE2-F 5′-ACAGTGACAGAGATGGGTGAAG-3′, HIPK2-ARE2-R 5′-GTGCCTTGGCTTTTCATCAAGG-3′. Left panel: the amount of material immunoprecipitated with anti-NRF2 (ab62352, Abcam) in DLD1 (WT) versus DLD1 NRF2-KO cells was compared. Right panel: we compared the amount of material immunoprecipitated with anti-NRF2 in DLD1 (WT) versus DLD1 NRF2-GOF cells. All data were normalised against the input lysates before enrichment by immunoprecipitation. Values are means plus s.d. from three independent experiments. (**e**) Luciferase gene reporter assay to identify functional AREs within the HIPK2 locus. The proximal HIPK2 promoter (−1 to −2000) and the HIPK2 intronic region (a 700 bp region spanning the identified ARE) were cloned into the basic-pGL3-luc vector, upstream and downstream of the luciferase gene, respectively. We used Nqo1-luc and mutARE-Nqo1-Luc as positive and negative controls, respectively.^56^ The indicated constructs were transfected into RL-34 cells (grown in DMEM containing 10% FBS) using Lipofectamine 2000. About 24 h after transfection, cells were treated either with vehicle or with 50 nm of TBE-31.^54^ Sixteen hours later, cells were lysed and their luciferase expression analysed using the Luciferase Assay System from Promega (Madison, WI, USA). The luciferase levels in vehicle-treated cells were set as 1. Values are means plus s.d. from five independent experiments.

Previously, we and others have shown that HIPK2 can affect redox balance and might regulate oxidative stress responses.^10, 48, 49^ As NRF2 is arguably the main transcription factor associated with oxidative stress responses, we wanted to test whether there was a link between NRF2 and HIPK2. To do so, we first used A549 cells (lung cancer cells that possess constitutively active NRF2 due to inactivating mutations on KEAP1^50^). We found that NRF2 knockout cells (NRF2-KO) have reduced protein and messenger RNA (mRNA) basal levels of HIPK2; conversely, NRF2 reconstitution restored both the protein and the mRNA levels of HIPK2 (Figure 1a). To monitor NRF2 activity, we used the prototypic NRF2 target gene NQO1 (Figure 1a; Supplementary Figure S1A). To our knowledge, there are no studies to date addressing the transcriptional regulation of HIPK2. We therefore confirmed the effect of NRF2 on HIPK2 mRNA levels, first by using different NRF2-KO cell lines (Supplementary Figures S1B and S1C) and second, by using various short hairpin RNA (shRNAs) against NRF2 (Supplementary Figure S1D). These results demonstrate that NRF2 regulates the steady-state levels of HIPK2.

In order to answer whether NRF2 activation is also able to induce HIPK2, we used two different approaches. In a pharmacological approach, we used two classical NRF2 activators, hydrogen peroxide (H_2_O_2_) and sulforaphane (SFN), which disrupt the KEAP1-dependent NRF2 degradation. We found that NRF2 activators increase both mRNA and protein levels of HIPK2 in H1299 cells (lung cancer cells with functional KEAP1), and that this induction requires NRF2 (Figure 1b). Of note, other KEAP1-proficient cell lines showed similar behaviour (Supplementary Figure S1E), and an overexpressed flag-tagged HIPK2 construct does not get stabilised by neither of NRF2 activators (Supplementary Figure S1F). Additionally, we used a genetic model: by deleting the KEAP1-binding motif within the endogenous NRF2, we produced cells that harbour a constitutively active NRF2 gain-of-function (GOF) mutant. Such GOF mutations are often found in tumours, and have been associated with malignancy.^43, 44, 51^ Thus, NRF2-GOF cells provide a physiologically relevant model of sustained NRF2 activation in malignant cells. We found that H1299 NRF2-GOF cells have elevated protein and mRNA levels of HIPK2 and NQO1 when compared with their WT counterparts (Figure 1c; Supplementary Figure S1G). The upregulation of HIPK2 observed in NRF2-GOF cells is not cancer cell type-specific (Supplementary Figure 1H). This model demonstrates that sustained NRF2 activation increases HIPK2 expression.

To identify the region(s) in the *HIPK2* gene that is responsible for its regulation by NRF2, we performed an *in silico* analysis of the *HIPK2* promoter to identify potential AREs that might be bound by NRF2. The minimal proposed consensus ARE sequence is TGASnnnGC (where S=C or G).^52^ As active AREs are often located close to the transcription start site, we focused on two ARE sequences we identified between −1 and −2000 bp within the *HIPK2* promoter, referred as ARE1 (at −246 bp: 5′-TGAGAGGGC-3′) and ARE2 (at −1794 bp: 5′-TGACTTAGC-3′). Additionally, Malhotra *et al.*^52^ using ChIP-seq in mouse cells, detected 1256 peaks as potential NRF2 binding sites. Among these, we identified a peak within a *HIPK2* intronic region; as this ARE sequence is conserved in humans (named intronic ARE: 5′-gTGACTCAGCg-3′), we analysed all three potential sites by studying NRF2 occupancy via ChIP-qPCR analyses. We used DLD1 cells to immunoprecipitate endogenous NRF2 and to interrogate its ability to bind to the three potential AREs. The analysis was performed by comparing the amount of material immunoprecipitated with anti-NRF2 antibodies in WT and NRF2-KO DLD1 cells (Figure 1d, left panel). Additionally, we compared the amount of material immunoprecipitated with anti-NRF2 or anti-IgG in DLD1 cells (Supplementary Figure S1I). Our results showed that at basal conditions NRF2 binds to the ARE1 and to the intronic ARE, but not to the more distal ARE2 sequence. Furthermore, we tested whether activation of NRF2 leads to an increase in its binding to the *HIPK2* locus. To do so, we used NRF2-GOF DLD1 cells as a model for NRF2-sustained activation and we compared them with DLD1 WT cells, and found that NRF2 activation leads to an enrichment of NRF2 bound to the *HIPK2* locus (Figure 1d, right panel). Finally, to address the potential functional relevance of the two identified sites, we used a luciferase-based genetic reporter assay. We cloned the proximal promoter of HIPK2 and the ARE-containing intronic region of HIPK2 upstream and downstream of the luciferase gene, respectively, and individually mutated the promoter ARE1 sequence and the intronic ARE sequence. We transfected these constructs into RL-34 cells (which are highly responsive to NRF2 inducers^53^) and tested their response to the NRF2 inducer TBE-31^54, 55^ (Figure 1e). We used plasmids containing either the promoter of *NQO1* fused to luciferase (WT) or the promoter of *NQO1* with a mutated ARE sequence fused to luciferase (MUT)^56^ as a positive and a negative control, respectively. Our results showed that while the ARE1 sequence situated within the *HIPK2* promoter does not control luciferase expression, the ARE sequence within the intronic region of *HIPK2* is responsible for the TBE-31-mediated induction of luciferase, highlighting the functional relevance of this ARE sequence.

Together, these results show that NRF2 regulates both basal and inducible levels of *HIPK2* at the transcriptional level via an intronic ARE sequence. The identification of a functional intronic ARE, although rare, has been recently reported for another NRF2 target gene.^57^ To our knowledge, this is the first demonstration of a transcriptional mode of regulation for *HIPK2*.

### HIPK2 supports NRF2 antioxidant response

**Figure 2:**
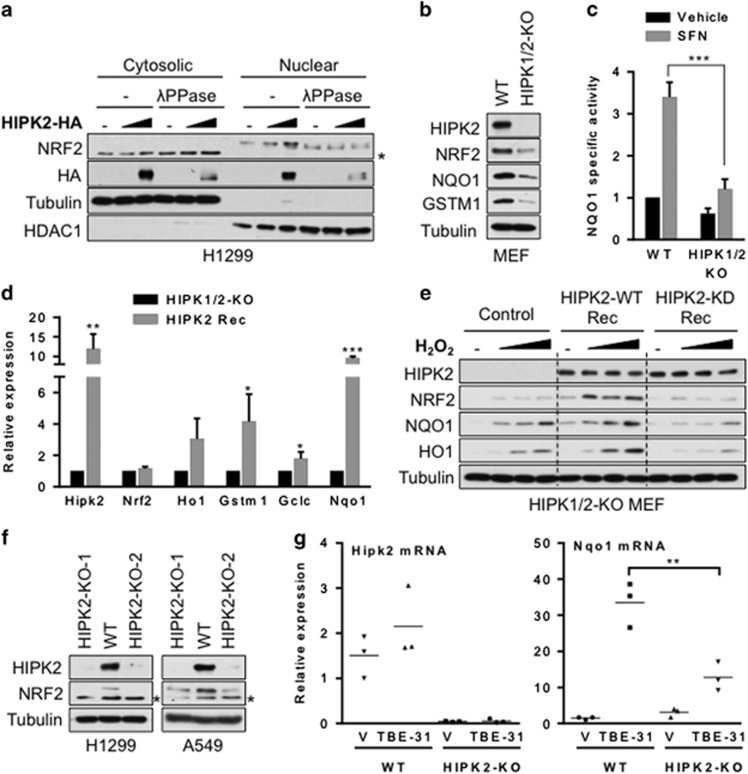
HIPK2 controls the NRF2 levels and shapes the NRF2 response. (**a**) H1299 cells were transfected using Lipofectamine 2000 with increasing concentrations of HA-tagged HIPK2.^10^ Cells were lysed 48 h after transfection and nuclear and cytosolic fractions were separated as previously described.^72^ Half of the protein extract was treated with Lambda phosphatase (New England Biolabs, Ipswich, MA, USA) for 1 h at 37 °C, boiled and analysed by western blotting using the indicated antibodies. Anti-HA (Y-11) was purchased from Santa Cruz Biotechnology. HDAC1 (H-51, Santa Cruz Biotechnology) and tubulin were used as markers for the nuclear and cytosolic fractions, respectively. The asterisk marks the position of an unspecific band. An empty gel lane was left between loading of the cytosolic and nuclear fractions. (**b**) WT or HIPK1/2-KO MEF cells^2^ (obtained from Dr Issay Kitabayashi (National Cancer Center Research Institute, Tokyo, Japan) grown in DMEM containing 10% fetal bovine serum (FBS) and 1% (w/v) penicillin/streptomycin were analysed by western blot for the levels of the indicated proteins using antibodies against HIPK2, NRF2,^31^ NQO1, GSTM1^31, 67^ and Tubulin. (**c**) WT or HIPK1/2-KO MEF cells were treated with vehicle (V) or with 3 μm Sulforaphane (SFN). After 7 h, cells were lysed and the specific activity of NQO1 was analysed as previously described.^73^ Values are means plus s.d. from three independent experiments. (**d**) Isogenic HIPK1/2-KO cells infected with empty vector or lentivirally reconstituted with HIPK2 (HIPK2 Rec) as described^10^ were analysed for the mRNA levels of Hipk2, Nrf2 and NRF2 target genes. The data were normalised using β-actin as an internal control. The mRNA levels of HIPK1/2-KO cells were set as 1. Values are means plus s.d. from three independent experiments. TaqMan probes were produced (mGstm1-Fw-primer 5'-CAAACCTGAGGGACTTCCTG-3', mGstm1-Rev-primer 5'-ATAGGTGTTGCGATGTAGCG-3', mGstm1-Probe 5'-CCGCTTCGAGGGCCTCAAGA-3') or obtained from Applied Biosystems: mNqo1 (Mm01253561_m1), mHipk2 (Mm00439329_m1), mActin (Mm00607939_s1), mHo1 (Mm00516005_m1), mGclc (Mm00802655_m1) and mNrf2 (Mm00477784_m1). (**e**) HIPK1/2-KO MEF were infected with empty vector (Control), or with virus encoding HIPK2 wild-type (HIPK2-WT Rec) or a kinase defective form of HIPK2 (HIPK2-KD Rec) as previously described.^10^ After puromycin selection, the isogenic cell lines were treated with increasing concentrations of hydrogen peroxide (20, 50, 75 μm). Eight hours later, cells were lysed and analysed for levels of the indicated proteins. (**f**) The endogenous *HIPK2* gene was knockedout by transfecting cells with pLentiCRISPR-v2 containing a guide RNA (gRNA) against the second exon of HIPK2 (5'-GCGAGGGCGACTATCAGC-3'). An additional gRNA (5'-GTGGTTCTTCAGGATCTTGA-3') was used to validate the results (data not shown). Control cells (referred as WT cells) were transfected with the empty pLentiCRISPRV2 vector. After 2 days of puromycin selection, cells were clonally selected by serial dilution, and positive clones were identified as previously described.^66^ Mutational gene changes in *HIPK2* were validated by sequencing of their genomic DNA. H1299 and A549 cells (WT) were compared with CRISPR-mediated H1299 and A549 HIPK2-KO cells. Protein levels of NRF2 and HIPK2 were analysed. Results obtained with two independent clones are shown. The asterisk marks the position of a non-specific band. (**g**) Hipk2^+/−^ mice crossed onto a C57BL/6 background (eight generations of backcrossing) were used.^3^ The heterozygous mice were intercrossed and their offspring, wild-type (WT) and Hipk2^−/−^ (HIPK2-KO), both male and female, were used for experiments at 8 weeks of age. Control mice (WT) or HIPK2 knockout mice (HIPK2-KO) were treated with the NRF2 inducer TBE-31: stock solution (3 mm) of TBE-31 was initially prepared in DMSO, and then diluted in phosphate-buffered saline at a 1:3 (v/v) ratio. The animals received TBE-31 (100 nmol/20 g body weight, 100 μl, i.p.) or the equivalent (100 μl) volume of vehicle (DMSO, 100 nmol/20 g body weight). After 16 h, livers were extracted and snap-frozen in liquid nitrogen. Samples were sectioned in ⩽30 mg fragments. RNA was extracted using RNeasy kit (Qiagen). Tissue was disrupted adding the appropriate volume of lysis buffer containing β-mercaptoethanol according to the manufacturer’s instructions and homogenised. Levels of Hipk2 and Nqo1 mRNA were evaluated. The data were normalised using β-actin as an internal control. The mRNA levels of one of the WT control mouse were set as 1. Values are means plus s.d. *n*=3 mice per group. The sample size was calculated using G*Power software v3.1^74^ to ensure *P*=0.05 at 95% power between treated samples in both genotypes. The differences between groups were determined by unpaired Student’s *t*-test. All animal experiments were carried out according to the in-house guidelines for the care and use of laboratory animals of the RIKEN, Yokohama Institute, Japan. Mice were assigned a random number, and treatments were blinded from the operator performing the data analysis.

Having demonstrated that HIPK2 is regulated by NRF2, we then studied whether HIPK2 affects NRF2-dependent responses. First, we found that HIPK2 overexpression promoted the accumulation of a nuclear, lambda phosphatase-sensitive form of NRF2 (in both endogenous and overexpressed NRF2) (Figure 2a; Supplementary Figure S2A). These results suggest that HIPK2 can activate NRF2 by promoting its nuclear accumulation, in a similar way as exposure to the oxidant H_2_O_2_ does (Supplementary Figure S2B). To test this possibility, we compared wild-type (WT) and HIPK2 knockout MEF cells and found that HIPK2-deficient cells had lower basal protein levels of NRF2 and the NRF2 target NQO1 and GSTM1 (Figure 2b), as well as an impaired induction of NQO1 after SFN treatment, as measured by enzyme activity (Figure 2c). Furthermore, HIPK2 reconstitution rescued (i) the basal mRNA levels of NRF2 target genes (Figure 2d) and their induction (in response to oxidants) (Supplementary Figure 2C) without affecting the mRNA levels of NRF2 itself, and (ii) the basal NRF2 protein levels, and its response to oxidants (measured by induction of NQO1 and HO1 levels upon exposure to H_2_O_2_) (Figure 2e). In these experiments, we used HIPK1/2-double knockout MEF cells to avoid potential compensation from HIPK1;^2, 3, 58^ similar results were obtained in single HIPK2 knockout cells (Supplementary Figure S2D). Interestingly, the effect of HIPK2 on NRF2 is kinase dependent, as a HIPK2 kinase-deficient mutant form (KD) did not rescue the basal NRF2 levels or the NRF2-mediated response (Figure 2e). However, based on our results we cannot distinguish between the effect of HIPK2 on NRF2 being direct or indirect.

To test the relevance of HIPK2 in the regulation of NRF2 in human cancer cells, we produced CRISPR-mediated HIPK2 knockout cells. We found that HIPK2 knockout decreased NRF2 levels in both H1299 and A549 lung cancer cells (Figure 2f). These results were confirmed in various cell lines using shRNAs against HIPK2 (Supplementary Figures S2E and S2F).

To test whether HIPK2 regulates NRF2 response *in vivo*, we analysed Hipk2, Nqo1 and Nrf2 mRNA levels from livers of wild-type and HIPK2 knockout mice treated with a single dose of the NRF2 inducer TBE-31. Compared to wild-type mice, HIPK2-deficient mice exhibited impaired induction of Nqo1 by TBE-31 (Figure 2g), without significantly affecting Nrf2 mRNA levels (Supplementary Figure S2G).

These results establish that HIPK2 contributes substantially to the NRF2-mediated responses both in cells and *in vivo*. Moreover, they highlight the potential role HIPK2 may play in ensuring a robust adaptive response against oxidative stress and xenobiotics. Of note, this new link between HIPK2 and oxidative stress responses is conserved throughout evolution, as a recent study demonstrated that in *Caenorhabditis* *elegans,* HPK-1 (the single homologue of HIPKs), confers resistance to oxidative stress.^59^

### Physiological relevance of the crosstalk between HIPK2 and NRF2

NRF2 is well-characterised as being cytoprotective in healthy tissue. In clear contrast, NRF2 increases resistance against a wide variety of chemotherapeutic drugs in malignant tissue. The fact that HIPK2 positively regulates NRF2 suggests that this HIPK2/NRF2 axis could represent a new pathway by which HIPK2 prevents tumour initiation. However, the existence of such axis provides a means by which HIPK2 might, via NRF2, play a hitherto unrecognised role in mediating survival of malignant cells upon challenge with chemotherapeutic drugs.

**Figure 3:**
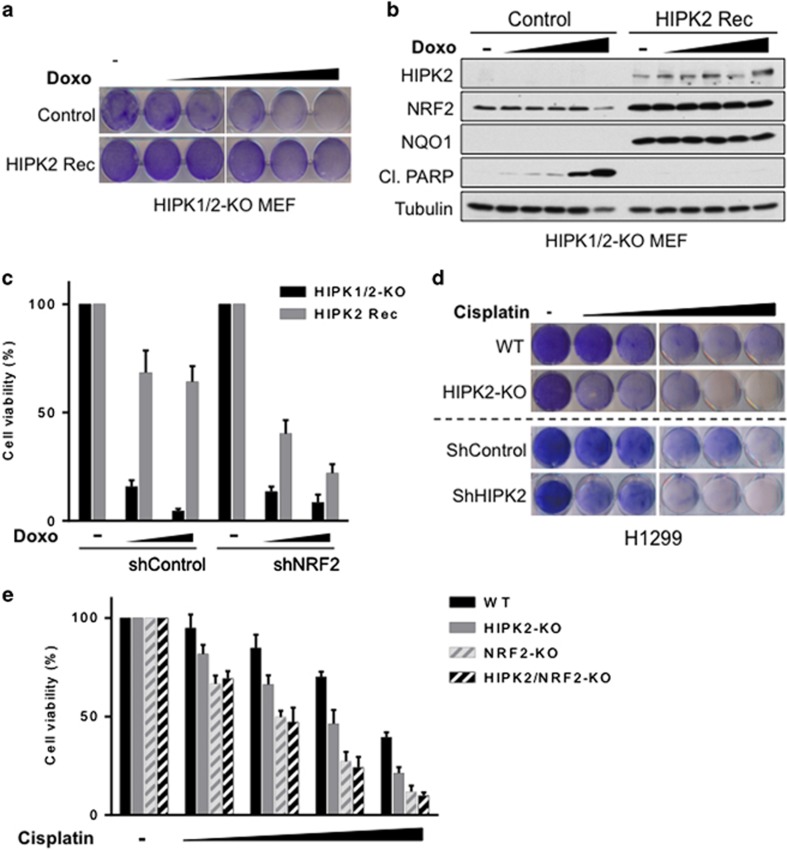
HIPK2 promotes cytoprotection via NRF2. (**a**) HIPK1/2-KO MEFs reconstituted with empty vector (Control) or with HIPK2 (HIPK2 Rec) were exposed to increasing concentrations (0.01, 0.05, 0.1, 0.5, 1 μg/ml) of Doxorubicin (Doxo) (Sigma-Aldrich, St Louis, MO, USA) for 4 h. After removal of the medium, the cells were washed and those that survived were further grown in complete DMEM. After 3 days, cells were washed with phosphate-buffered saline and fixed with ice-cold methanol for 10 min, stained with a crystal violet solution (0.5% crystal violet, 25% ethanol) for 20 min and rinsed with water to remove the excess of dye. (**b**) HIPK1/2-KO reconstituted with empty vector (Control) or with HIPK2 (HIPK2 Rec) were exposed to increasing concentrations (0.01, 0.05, 0.1, 0.5, 1 μg/ml) of Doxo. After 12 h, cells were analysed for the levels of the indicated proteins by western blot. Antibodies against cleaved PARP (cl.PARP, #9544) were obtained from Cell Signalling (Danvers, MA, USA). In the gels there is an empty lane between both cell lines. (**c**) HIPK1/2-KO MEF and HIPK1/2-KO MEF reconstituted with HIPK2 were infected with virus carrying a non-targeting shRNA (shControl; SIGMA Mission shRNA PLKO.1-puro) or an shRNA against NRF2 (SIGMA Mission shRNA. TRCN0000007555, targets 5'-AAAAGCTCCTACTGTGATGTGAAAT-3'). Equal number of cells were seeded in a 96-well plate and treated with doxorubicin (0.5, 1 μg/ml) for 4 h. After removal of the medium, cells were washed once and those that survived were further grown in complete DMEM for 3 days. At that point Alamar Blue was added to the media and incubated for 1–3 h at 37 °C according to the manufacturer’s instructions. The resulting fluorescence was quantified using a plate reader. Values are means+s.d. from three independent experiments. Similar results were obtained using additional shNRF2 (SIGMA Mission shRNA. TRCN0000054658, targets 5'-CCAAAGCTAGTATAGCAATAA-3') (Data not shown). (**d**) H1299- (WT) or CRISPR-mediated H1299 HIPK2-KO cells (upper panels), or H1299 infected with virus carrying a non-targeting shRNA (shControl) or an shRNA against HIPK2 (shHIPK2) (SIGMA Mission shRNA, TRCN0000023014, targets 5'-CACCCATGATTCAGAATAAT-3') (lower panels) were exposed to increasing concentrations of cisplatin (2, 4, 8, 16, 20 μg/ml) for 12 h followed by a medium exchange. The surviving cells were further grown for 3 days and stained with crystal violet. (**e**) H1299- (WT), CRISPR-mediated H1299 HIPK2-KO, NRF2-KO or double NRF2/HIPK2-KO cells were seeded in 96-well plates and exposed to increasing concentrations of cisplatin (2, 4, 8,16 μg/ml) for 8 h followed by a medium exchange. The surviving cells were further grown for 3 days and Alamar Blue was added to the media. Cell viability was determined following absorbance measurement. Values are means plus s.d. from four independent experiments.

To address whether HIPK2 affects cell responses to chemotherapeutic drugs via NRF2, we used two different approaches. First, we reconstituted HIPK1/2-KO cells with HIPK2 (or with an empty vector) and exposed both isogenic cell lines to increasing concentrations of the commonly used chemotherapeutic drug doxorubicin. We found that, compared to HIPK1/2-KO MEFs, cells reconstituted with HIPK2 exhibited higher cell viability (Figure 3a) and a striking reduction of apoptosis (measured by PARP cleavage) in response to doxorubicin, correlating with higher levels of NRF2 and NQO1 (Figure 3b). In full agreement with the apoptosis data, HIPK2-reconstituted cells were more resistant to doxorubicin (measured by cell viability), and this resistance was significantly reduced by NRF2 knockdown (Figure 3c). Second, we knockedout HIPK2, NRF2 or both genes in H1299 lung cancer cells, and measured their sensitivity to cisplatin compared with wild-type cells. HIPK2 knockout (or knockdown) increased the sensitivity to cisplatin as seen by a colony-formation assay (Figure 3d). Furthermore, whereas knockout of NRF2 sensitised cells to cisplatin, a double knockout of both NRF2 and HIPK2 did not increase drug sensitivity further as shown by a cell viability assay (Figure 3e).

These results confirm that HIPK2 can promote cell survival upon challenge with chemotherapeutic drugs; this, together with the involvement of the well-established pro-survival factor NRF2 makes a strong case for the idea that, by activating NRF2, HIPK2 could support cancer cell survival.

## Conclusions

**Figure 4:**
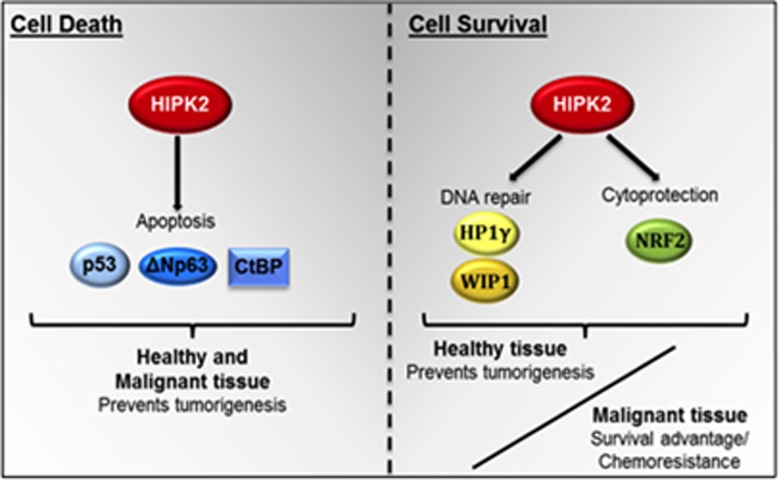
Schematic representation of the proposed differential role of HIPK2 in healthy and malignant tissue. HIPK2 pro-apoptotic role can promote clearance of damaged cells in healthy tissue and also the elimination of malignant cells in response to cancer therapeutics. In contrast, HIPK2 pro-survival role might help cancer avoidance by preventing DNA mutations in healthy tissue, but it might also protect malignant cells against genotoxic insults.

Herein, we demonstrate for the first time that HIPK2 is regulated at the transcriptional level and that *HIPK2* is an NRF2 target gene. This is a notable finding because most HIPK2 regulatory mechanisms described to date rely on posttranslational modifications and thus, our results add an extra regulatory layer to the control of HIPK2 activity. Additionally, our data place HIPK2 as a critical kinase, shaping the NRF2 response. This is important in the cancer biology field for two reasons: First, our discovery reveals HIPK2 to be a common apical regulator of two major stress regulated pathways, NRF2 and p53, and thus a decisive factor controlling cancer cell fate by being coupled to both cell death and cell survival. It is important to highlight that although HIPK2 can protect healthy tissue against tumour initiation by promoting both DNA repair^17, 22^ and cytoprotection (shown in this study), activation of the same pro-survival pathways in malignant tissue could lead to aberrant cell survival and enhanced chemoresistance (see model in Figure 4), particularly when apoptotic pathways are impaired (that is, in the absence of a functional p53). In this context, it will be important in the future to address whether HIPK2 plays opposing roles depending on the stages of tumour development (for example, preventing initiation but accelerating progression) as has also been proposed for NRF2.^60^ Interestingly, HIPK2 also controls the levels of Notch1,^61^ a well-known factor with a dual role in cancer, which in common with NRF2, can act as both tumour suppressor and oncogene, depending on the context,^62, 63, 64, 65^ adding strength to the idea of a context-dependent role for HIPK2 in cancer. Second, our data suggest that inhibition of HIPK2 could be a plausible mechanism by which the NRF2 pathway could be suppressed, thereby providing a new strategy to overcome NRF2-associated resistance to therapies in malignant cells. It is recognised that aberrant-sustained activation of NRF2 can promote chemoresistance and radioresistance, and therefore, inhibition of NRF2 in these settings should increase the efficacy of anticancer therapies.

In summary, our results provide new information supporting the already well-established tumour suppressor role of HIPK2, and also could explain how under certain conditions (for example, cancer cell chemoresistance due to upregulated NRF2) HIPK2 might provide cancer cells with a survival advantage.
