# Supplementary material for: Crosstalk between NRF2 and HIPK2 shapes cytoprotective responses
Source: Oncogene. 2017 Jul 10;36(44):6204–12. doi: 10.1038/onc.2017.221 (PMC5641449; doi:10.1038/onc.2017.221)
Supplement: Supplementary Material [file onc2017221x1.docx]

Supplementary Material

Supplementary Figure legends

Supplementary Figure 1.

(S1A) TaqMan analysis of NQO1 mRNA levels in A549 control cell lines (WT) compared with NRF2-KO cells or NRF2-KO cells reconstituted with NRF2 (NRF2 Rec). The data were normalized using β-*actin* as an internal control. The mRNA levels in WT cells were set as 1. Values are means plus SD from three independent experiments

(S1B) TaqMan analysis of HIPK2 and NQO1 mRNA levels in HeLa, H1299 and DLD1 control cell lines (WT) compared with NRF2-KO cells. The data were normalized using β-*actin* as an internal control. The mRNA levels in WT cells were set as 1. Values are means plus SD from three independent experiments

(S1C) KEAP1/NRF2-KO MEFs obtained from Prof Masayuki Yamamoto (Tohoku University, Japan)[^1^](#_ENREF_1) were infected with empty vector (Control) or with a vector encoding for NRF2 (NRF2 Rec). After puromycin selection cells were analysed by western blot for the levels of the indicated proteins (*Left Panel*), and mRNA levels of NRF2 and HIPK2 were analysed by TaqMan (*Right Panel*). The data were normalized using β-*actin* as an internal control. The mRNA levels of cells infected with empty vector were set as 1. Values are means plus SD from three independent experiments.

(S1D) A549 cells were infected with virus carrying a non-targeting shRNA (shControl), or shRNAs against NRF2: shNRF2-a (TRCN0000007558, targets CCGGCATTTCACTAAACACAACTC) and shNRF2-b (TRCN0000007555, targets AAAAGCTCCTACTGTGATGTGAAAT). After puromycin selection, mRNA levels of NRF2, HIPK2 and NQO1 were analysed by TaqMan. The data were normalized using β-*actin* as an internal control. The levels of mRNA in cells infected with shControl were set as 1. Values are means plus SD from three independent experiments.

(S1E) WT or NRF2-KO HeLa cells were treated with vehicle, 100 μM hydrogen peroxide (H_2_O_2_) or with 3 μM Sulforaphane (SFN). After three hours, mRNA and proteins were extracted. In the *Left Panel* the levels of HIPK2 mRNA were analysed. The mRNA levels in vehicle treated WT cells were set as 1. Values are means plus SD from five independent experiments. In the *Right Panel* the levels of the indicated proteins were analysed by western blot.

(S1F) HeLa cells were transfected with HIPK2-Flag. 36 hours later, cells were treated with vehicle, 100 μM of Hydrogen peroxide (H_2_O_2_) or 3 μM of Sulforaphane (SFN). After three hours, cells were lysed and analysed by western blot for the levels of the indicated proteins. The asterisk marks the position of an unspecific band.

(S1G) TaqMan analysis of NQO1 mRNA levels in H1299 control cells (WT) compared with CRISPR-mediated NRF2- Gain-of-function (NRF2-GOF) H1299 cells. Results obtained using two independent NRF2-GOF clones are shown.

(S1H) TaqMan analysis of HIPK2 and NQO1 mRNA levels in DLD1 control cells (WT) compared with CRISPR-mediated NRF2- Gain-of-function (NRF2-GOF) DLD1 cells.

(S1I) ChIP analysis of NRF2 occupancy within the HIPK2 proximal promoter (up to -2000bp) and intronic region. We compared the amount of material immunoprecipitated with anti-NRF2 or with IgG (SantaCruz Biotechnologies) by RT-qPCR in DLD1 cells. We used specific primers for each of the potential NRF2-binding sites within the HIPK2 locus and for the previously characterised ARE within the NQO1 promoter (positive control).

Supplementary Figure 2.

(S2A) HeLa cells were grown in DMEM containing 10% FBS. Equal number of cells were transfected with NRF2-HA (a gift from Dr Donna D. Zhang, University of Arizona, USA) in combination with increasing concentrations of Flag-HIPK2 (or with empty vector). Cells were lysed 48 hours after transfection, and nuclear and cytosolic fractions were separated. Half of the protein extract was treated with Lambda phosphatase for 1 hour at 37ºC, boiled and analysed by blotting with the indicated antibodies. Lamin B (C-20, Santa Cruz Biotechnology) and tubulin were used as markers for nuclear and cytosolic fractions respectively. In the gels there was an empty lane between the cytosolic and nuclear fractions.

(S2B) H1299 cells were treated with increasing concentrations of hydrogen peroxide (50 and 75 μM). After four hours, cells were lysed and nuclear and cytosolic fractions were separated. Samples were treated with and without Lambda phosphatase for 1 hour at 37ºC, boiled and analysed by western blotting using the indicated antibodies. HDAC1 and tubulin were used as markers for the nuclear and cytosolic fractions respectively. The asterisk marks the position of an unspecific band. An empty gel lane was left between loading of the cytosolic and nuclear fractions.

(S2C) Isogenic HIPK1/2-KO cells infected with empty vector or lentivirally reconstituted with HIPK2 (HIPK2 Rec) were treated with vehicle or with 100 μM hydrogen peroxide (H_2_O_2_) for two hours (for Hipk2, Nrf2, Ho1 and Gclc) or eight hours (for Nqo1). Levels of the indicated genes were analysed by Taqman. The data were normalized using β-*actin* as an internal control. The mRNA levels of HIPK1/2-KO cells were set as 1. Values are means plus SD from three independent experiments.

(S2D) Primary wild-type and HIPK2-KO MEFs were derived from wild-type or HIPK2-KO[^2^](#_ENREF_2) littermate embryos at 12.5 dpc. Equal number of WT or HIPK2-KO MEFs were analysed by western blot for the levels of the indicated proteins.

(S2E) HeLa cells were transfected with empty vector (shControl) or with vectors coding for shRNA against HIPK2: shHIPK2-a (TRCN0000433047, targets CCCACAGCACACACGTCAAATC), shHIPK2-b, (TRCN0000361235, targets AATCCCGAAGTCTCCATACTAAACT), shHIPK2-c (TRCN0000414399, targets GTTCCTGGGTTGGCCGTTATATC), shHIPK2-d (TRCN0000023014, targets CACCCATGATtCAGAATAAT). After puromycin selection, surviving cells were analysed for the levels of the indicated proteins. The asterisk marks the position of an unspecific band.

(S2F) H1299 and A549 cells were infected with virus carrying a non-targeting shRNA (shControl) or an shRNAs against HIPK2 (shHIPK2-d). After puromycin selection, equal number of surviving cells were analysed for the levels of the indicated proteins. Tubulin was used as a loading control. The asterisk marks the position of an unspecific band.

(S2G) Control mice (WT) or HIPK2-knockout mice (HIPK2-KO) were treated with the NRF2 inducer TBE-31 (100 nmol/20g body weight) or with DMSO as vehicle (V). After 16h, livers were extracted and snap-frozen in liquid nitrogen. mRNA was extracted from the frozen livers and the levels of NRF2, were evaluated. The data were normalized using β-*actin* as an internal control. The mRNA levels of one of the WT control mouse were set as 1. n=3 mice per group. The differences in the NRF2 levels between groups were not statistically significant.

Supplementary References

1. Wakabayashi N, Itoh K, Wakabayashi J, Motohashi H, Noda S, Takahashi S, et al. Keap1-null mutation leads to postnatal lethality due to constitutive Nrf2 activation. Nat Genet. (2003) Nov;35(3):238-45.

2. Isono K, Nemoto K, Li Y, Takada Y, Suzuki R, Katsuki M, et al. Overlapping roles for homeodomain-interacting protein kinases hipk1 and hipk2 in the mediation of cell growth in response to morphogenetic and genotoxic signals. Mol Cell Biol. (2006) Apr;26(7):2758-71.
